# Clinical Utility of Transforaminal Epidural Steroid Injection: A Systematic Review and Meta-Analysis to Study the Predictors of Favorable Surgical Outcomes

**DOI:** 10.7759/cureus.99325

**Published:** 2025-12-15

**Authors:** Purushottam Kumar, Suyash Singh, Priyanka Priyanka, Kurvatteppa Halemani, Anirudh Mukherjee, Rajat S Das

**Affiliations:** 1 Neurosurgery, All India Institute of Medical Sciences Raebareli, Raebareli, IND; 2 Microbiology, All India Institute of Medical Sciences Raebareli, Raebareli, IND; 3 Nursing, Sanjay Gandhi Postgraduate Institute of Medical Sciences, Lucknow, IND; 4 Internal Medicine, All India Institute of Medical Sciences Raebareli, Raebareli, IND; 5 Anatomy, All India Institute of Medical Sciences Raebareli, Raebareli, IND

**Keywords:** epidural steroid injection (esi), favorable surgical outcomes, fluoroscopy, low back pain (lbp), prognostic factors

## Abstract

The study analyzes current data based on clinical, radiological, and procedural parameters in order to determine predictors of effective transforaminal epidural steroid injection (TFESI) results. The systematic review and meta-analysis (PROSPERO: CRD42024497170) implemented PRISMA and Cochrane guidelines for its methodology. The search of multiple databases during the last 20 years resulted in 30 studies that met the criteria for evaluating TFESI outcome predictors. The research team conducted data extraction and quality assessment, followed by random-effects proportion-based meta-analysis to present findings through tables and forest plots, and risk-of-bias analysis. The meta-analysis evaluated 56 prognostic factors for TFESI outcomes. Findings showed that better outcomes occurred in younger patients who had a shorter symptom duration, lower body mass index, and no previous spine surgery history. Likewise, certain disc herniation locations, low-grade nerve root compression, and contrast distribution patterns were shown to be imaging markers for improved results. Poor treatment outcomes were associated with anatomical variations, facet tropism, and severe central stenosis. Overall evidence was heterogeneous, although electrodiagnostic results and procedural accuracy also had an impact. The study identified a number of factors that influence lumbar transforaminal epidural steroid injection procedure success rates. Shorter duration of symptoms, no prior surgery, and certain MRI findings were more dependable; however, demographic considerations indicated inconsistent results.

## Introduction and background

Low back pain (LBP) with or without radiculopathy is a prevalent cause of disability and healthcare burden worldwide, affecting nearly 80% of adults at some point in their lives [1,2]. It represents a major public health burden with significant socio-economic implications, including reduced quality of life, loss of work productivity, and increased healthcare utilization [3]. Among the various etiologies of LBP, lumbar radiculopathy, often caused by disc herniation or foraminal stenosis, is the most common condition. These herniations are characterized by radiating leg pain, paresthesia, or weakness, resulting from nerve root compression or inflammation [4]. Management of lumbar radiculopathy typically spectrums from conservative therapy, including pharmacological agents (e.g., NSAIDs, muscle relaxants), physical therapy, to activity modification [5]. While a majority of patients improve with non-invasive approaches, a subset continues to experience persistent pain and functional limitation, prompting consideration of interventional procedures. Among these management strategies, epidural steroid injections (ESIs) have been widely adopted for both diagnostic and therapeutic purposes. ESIs are classified into three major approaches: caudal, interlaminar, and transforaminal [6].

Among the available epidural approaches, transforaminal epidural steroid injection (TFESI) is regarded as the most targeted and effective technique, especially for unilateral radiculopathy [7]. TFESI allows precise delivery of corticosteroids and local anesthetics into the anterior epidural space, near the affected nerve root, resulting in enhanced anti-inflammatory action and pain relief [8], providing both diagnostic and therapeutic benefits [9]. Multiple studies have demonstrated superior outcomes of TFESI in terms of pain relief, functional improvement, and avoidance or postponement of surgery compared to interlaminar or caudal routes [10,11]. Moreover, image-guided (fluoroscopic or CT) TFESI enhances safety and accuracy, reducing the risks of inadvertent dural puncture or vascular injection [12].

Despite the widespread use of TFESI, the clinical efficacy of TFESI remains variable, with response rates ranging from 40% to 80% [13]. While some experience substantial and sustained relief, others report minimal or transient benefits [14]. This heterogeneity in outcomes has prompted extensive investigation into the predictive factors that might determine a favorable response to TFESI, especially in the context of avoiding or delaying surgical intervention. These predictors include demographic variables (e.g., age, gender), clinical characteristics (e.g., duration of symptoms, pain distribution), radiological findings (e.g., degree of nerve root compression, contrast spread pattern), and procedural aspects (e.g., level of injection, type and dose of corticosteroid used) [15-17].

Several original research studies have attempted to explore the predictors of favorable outcomes following TFESI, such as younger age, shorter symptom duration, presence of disc herniation (vs. spinal stenosis), higher baseline pain scores (measured via the visual analog score or numerical rating scale), and imaging findings like nerve root compression or enhancement on MRI [18-20]. However, the existing literature is fragmented and individually explores these variables, but their conclusions are often conflicting or limited by small sample sizes, lack of control groups, and heterogeneity in methodology. Moreover, there is a lack of comprehensive synthesis that quantitatively evaluates the predictors of successful surgical outcomes following TFESI. Given the rising demand for precision medicine and personalized interventional approaches, understanding which patients are most likely to benefit from TFESI is critical for optimizing clinical decision-making and resource utilization [21].

Therefore, this systematic review and meta-analysis aim to rigorously evaluate the available evidence on predictive factors for favorable surgical outcomes after TFESI. By synthesizing high-quality data from multiple studies, this work seeks to offer clinically actionable insights that can inform patient selection, improve outcomes, and reduce unnecessary surgical interventions.

## Review

Methodology

This systematic review and meta-analysis were registered with PROSPERO (CRD42024497170) and complied with the Preferred Reporting Items for Systematic Review and Meta-Analysis (PRISMA) and the Cochrane Collaboration guidelines [22]. The search strategies were carried out after fulfilling review criteria and compiled with the Population, Intervention, Control, and Outcome (PICO) format. The population comprised patients with (low back pain[Mesh] OR back pain [Mesh] OR non-invasive procedure[Mesh] OR epidural steroid injections[Mesh] OR transforaminal lumbar epidural steroid injection[Mesh] OR Epidural injection [Mesh] transforaminal block [Mesh] OR clinical efficacy of Transforaminal Epidural Steroid Injection [Mesh] OR nerve root block [Mesh] OR nerve root compression [Mesh] OR nerve block [Mesh] OR Radiculopathy [Mesh] OR Spondylosis [Mesh]) AND (Radicular back pain [tiab] OR radicular low back pain [tiab] OR low back pain[tiab] OR back pain [tiab] OR non-invasive procedure[tiab] OR epidural steroid injections[tiab] OR transforaminal lumbar epidural steroid injection[tiab] OR Epidural injection [tiab] transforaminal block [tiab] OR clinical efficacy of Transforaminal Epidural Steroid Injection [tiab] OR nerve root block [tiab] OR nerve block [tiab] OR Radiculopathy [tiab] OR Spondylosis [tiab] OR efficacy[tiab] OR Predict*[tiab] OR effect*[tiab] OR prognos*[tiab] OR affect[tiab] OR success[tiab] OR outcome[tiab] OR associat*[tiab] OR predictive factor[tiab]). The review comprises prospective or retrospective RCTs published in the English language between January 2005 and May 2025 (20 years).

The search was conducted from various databases, including PubMed, Clinical Key, CINAHL Cochrane Library, Web of Science, EMBASE, Google, and Google Scholar. The keywords, Medical Subject Headings (MeSH), Table/Abstract (tiab), were developed and tailored with a PICO statement and entered into a search engine. Further, secondary studies were tracked through citations of primary studies. The two authors independently screened, and disagreements were resolved by a third author through mutual agreement. The retrieved data were presented in the following headings: name of the author, publication year, research design, sample size, studied factors, predictive factor, inclusion and exclusion criteria, instruments used, and study outcomes.

Studies were included based on original research articles including randomized controlled trials (RCTs), prospective and retrospective cohort studies, and case-control studies, adults (≥18 years) diagnosed with lumbar radiculopathy or related lumbar spine conditions who underwent TFESI, transforaminal lumbar epidural steroid injection performed under fluoroscopic or CT guidance, studies that reported predictors of favorable surgical outcomes following TFESI, such as pain relief (VAS or NRS has been used to measure the pain), functional improvement, or reduced need for surgery, articles published in English. Exclusion criteria include studies involving animals or cadavers, case reports, editorials, conference abstracts, and narrative reviews, studies not reporting surgical outcomes after TFESI, studies that did not meet the success criteria of ≥50% improvement in pain, and studies without clear documentation of predictive variables.

**Figure 1:**
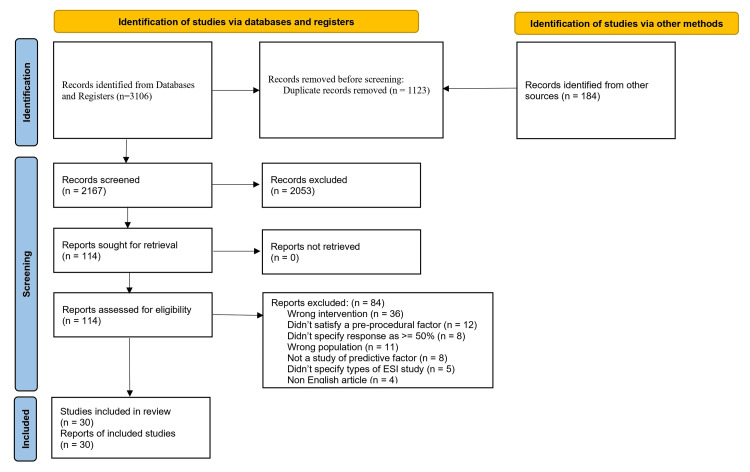
PRISMA diagram outlining the search strategy PRISMA: Preferred Reporting Items for Systematic Reviews and Meta-Analyses

During the first stage, 3290 studies were found from various databases and printed materials. Out of these, 1123 duplicate studies and further 2053 records were excluded after screening of the title and abstract. The remaining 114 full trials were screened in depth; again, 84 articles failed to meet the review criteria due to inadequate methodology and statistical information. Finally, 30 studies were included in the systematic review and meta-analysis after meeting the review criteria. Figure 1 depicts this research selection process.

**Figure 2:**
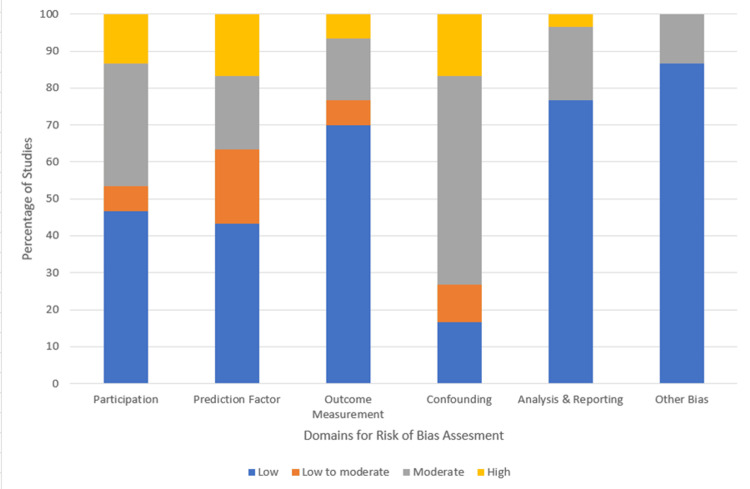
Risk of bias assessment summary (in %)

The quality of the primary studies was assessed using the Cochrane Collaboration’s “risk of bias tool”. The tool consists of the following headings, namely study participation, predictive factors, outcome measurements, study confounding, reporting and statistical analysis, and other bias. After evaluating the quality of studies were categorized into low risk, unclear (low to moderate, moderate), and high risk (Figure 2).

The retrieved data are summarized and presented in Tables 1, 2. The weightage of studies was calculated using the proportion-based meta-analysis, where the proportion of studies indicating a positive correlation for each predictor was calculated. The percentage of supporting studies (k/n) for each predictor was summed with 95% confidence intervals utilizing the Wilson technique. This method offers a comprehensive measure of consistency across studies based on vote counting. Overall analysis was done using RevMan software (version 5.4.1) [23,24].

**Table 1:** Summary of studies evaluating various predictive factors of TFESI success TFESI: Transforaminal epidural steroid injection; ESI: epidural steroid injection; LDH: lumbar disk herniation

S.No	Study (Author, Year)	Type of Study	Sample Size	Level	Predictors Evaluated	Significant Predictors	Outcome Measures	Follow-Up Duration
1	Cyteval et al., 2006 [25]	Prospective	228	L3 to S1	Age, sex, conflict location, symptom duration, VAS, cause of pain	Symptom duration (average 3.04 months)	Pain relief excellent in 19.7%, good in 21%, fair in 19.7%	2 weeks, 1 year
2	Choi et al., 2007 [26]	Retrospective	68	L4-L5, S1	Age, sex, type of disc degeneration (protrusion, extrusion, sequestration), hydration (signal intensity), location (central, left/right central, subarticular, foraminal, or extraforaminal), volume of HIVD, grade of nerve root compression, and spinal stenosis	Location of HIVD (centrally located) and grade of nerve root compression (lower grade)	Pain satisfaction score >2, and >50% VAS score reduction	7 days, 24 months
3	Park et al., 2008 [27]	Retrospective	75	L4-L5, L5-S1, S1	Age, Gender, Duration of symptoms, VAS, ODI, Beck anxiety inventory (BAI), Beck depression inventory (BDI), cause of radiculopathy (stenosis, HIVD), TFESI approach (1 level, OR 2 level)	Two-level injection has significantly better outcome over single-level injection. ODI and VAS decreased significantly in both groups (1 level and 2 level)	≥65% had satisfactory result	2 weeks
4	Ghahreman et al., 2011 [28]	Retrospective	71	L2-3 to L5-S1	Age, sex, duration of symptoms, pain severity, presence of neurological symptoms, sensory deficit, abnormality of reflex, motor deficit, level and side of nerve compression, morphology of disc displacement, location of the herniation, end plate osteophytes, facet hypertrophy, ligamentum flavum hypertrophy, spondylolisthesis, foraminal narrowing	Low-grade nerve root compression	≥50% reduction in VAS score	1 months
5	Paidin et al., 2011 [29]	Retrospective	81	L3-4, L5-S1, S1	Age, sex, level injected, injection type, contrast pattern type, grade of nerve root compression	Type I and III contrast pattern is a significant factor in pain reduction	NRS	15 minutes, 2 weeks, and 2 months
6	Lee et al., 2012 [30]	Retrospective	56	L1-2 to L5-S1	Age, sex, duration of symptoms, additional extra epineural injection	TES is an effective tool, additionally an extra epineural injection may be a predictor	≥50% reduction in VAS score	2 weeks
7	Iversen et al., 2011 [31]	Prospective	116	L2-3 to L5-S1	Sex, age, BMI, nerve root compression	Female sex and BMI <30 correlated with better response	VAS reduction ≥ 50%	6 weeks
8	Lee et al., 2013 [32]	Retrospective	149	L1 to S1	Age, Sex, symptom duration, pattern of symptom attack (initial vs recurrent), HIVD type, HIVD zone, HIVD volume, high signal in HIVD, relation of HIVD to nerve root, corner change, Modic change, disc height loss, grade of disc degeneration, and poster osteophyte	HIVD in either or both the foraminal and extraforaminal zone	No pain OR ≥ 50% reduction in VAS score	2 weeks, 2 years
9	McCormick, Zachary et al., 2014 [33]	Retrospective	188	L2-3 to L5-S1, S1, Bilateral injections	Age, sex, smokers, unemployed, worker’s compensation benefits, duration of pain at presentation (mo), follow-up time, VAS pain score, disk herniation, stenosis (central, and foraminal), spondylolisthesis, other, level of injection (L2-3, L3-4, L4-5, L5-S1, S1 foramen, bilateral injection), preinjection depression	Patient with greater preinjection VAS or McGill Pain inventory score, or disk herniation had experienced more pain relief. Lack of worsening of pain with walking	>50% pain reduction	2 weeks
10	Maus et al., 2016 [34]	Retrospective	516		Age, sex, pain type, level, steroid type, compressive lesion, compressive grade, tandem lesion, NRS, nature of compressive lesion, degree of neural compression	Disc herniation, single lesion had better significance	Reduced NRS ≥50%, or R-M by 40%	2 weeks, and 2 months
11	Tecer et al., 2017 [35]	Retrospective	59		Age, sex, pain severity, symptom duration, herniation type, herniation location, high intensity zone (HIZ), nerve root impingement (NRI)	Patient with HIZ at the second week and patients with NRI at the third month had great improvement	≥50% reduction in VAS score	2 weeks, and 3 months
12	Kanna et al., 2018 [36]	Prospective	91	L3-4, to L5-S1	Age, sex, duration of symptoms, side of pain, level of lesion, presence of inciting event, presence of back pain, type of job (manual laborer, white collared, home maker), BMI, presence of sensory symptoms along the affected nerve root(subjective paresthesia and objective anesthetic zones), VAS score for leg pain, presence of previous similar episodes, smoking, comorbidities, straight leg raise test positivity, and ODI score, level of disc prolapse, position of disc, the type disc herniation (bulge, protrusion, extrusion, sequestration), Pfirrmann’s grade of the affected disc, and the presence of LS transitional vertebra at the level , classification of radiculogram (arm, arrow, linear, and splash)	Sensory symptoms (P=.01), higher mean preinjection ODI score (P=.02), higher mean postinjection ODI score at 3 weeks (P=0.004), having a white-collared office job (P=.01), and LS transitional segment (P-.00005) and splash pattern of radiculogram (P=.005)	≥75% had good pain relief	1 year
13	Miskin et al., 2018 [37]	retrospective	68	L2-L3 to L5-S1	Needle position (posterolateral, central, anteromedial), and direction of contrast flow (central, peripheral), degree of neural foraminal stenosis, volume of contrast injection, pain score (NRS)	No significant association with needle position and degree of foraminal stenosis	≥60% reduction in pain score	Immediate post-procedure
14	Chang et al., 2018 [38]	Prospective	60	L3/L4/L5	Age, sex, pain severity (NRS), pain duration, site of pain, injection level	Mild to moderate degree of LFSS	≥50% reduction in NRS score	1, 2, 3 months
15	Park et al., 2019 [39]	retrospective	218	L2-3 to L5-S1, S1	Age, sex, diabetes, hypertension, trauma or sprain, previous spine surgery, duration of symptoms, neurological claudication, SLR test, motor and sensory exam, no. of herniated nucleus pulposus, nerve root compression, canal stenosis, electrodiagnosis	Symptoms duration (effective for actue symptoms), PSW/Fib related to limb muscle LS radiculopathy by EDx, absence of spine surgery,	61% short term pain relief	2-4 weeks
16	Celenlioglu et al., 2019 [40]	Prospective	112	L3–4, L4–5, or L5-S1	Age, sex, BMI, symptom duration, medication used before the treatment, duration of medication use, history of physical therapy, NRS, modified ODI, BDI, type of LDH, localization, facet tropism, focal or broad base formation, Modic changes	Facet tropism reduces the success rate	≥50% reduction in NRS score	1 hour, 3 weeks, 3 months
17	Sencan et al., 2020 [41]	retrospective	219	L4-5, L5-S1, S1	Age, sex, BMI, duration of symptoms, injection levels, pain score, grade of nerve root compression, sciatica	LDH-induced sciatica patients	≥50% reduction in VAS score	1 hours, 3 months
18	Bahar-Ozdemir et al., 2020 [42]	prospective	103	L3 to S1, L2-3 to L5-S1	Age, BMI, HADS (depression and anxiety level), SSAS, injection side, injection level	High preinjection depression	≥50% reduction in ODI score	1 hours, 3 months
19	Jin et al., 2022 [43]	Prospective	280	L4-5	Age, sex, contrast spread pattern, fat infiltration, disc degeneration	RD approach in patients with severe central and foraminal spinal stenosis → better outcome	VAS and ODI	4 weeks
20	Leblebicier et al., 2021 [44]	Prospective	46	L5	Age, sex, height, weight, BMI, duration of symptoms, side of root compression (left or right), ODI, BDI, VAS, PM score, L5 PM score	Lower score of L5 PM score,	≥80% reduction in VAS score	3 months
21	Sencan et al., 2021 [45]	Prospective	61	L5-S1, S1	Age, sex, education status, pain severity, ODI, BDI, duration of radiculopathy, Douleur Neuropathique 4 Questionnaire (DN4) for NP	NP is a risk factor that adversely affects the TFESI success	DN4 score, NRS	1 hour, 3 weeks, 3 months
22	Sencan et al., 2023 [46]	Prospective	64	L5, S1	Age, sex, BMI, NRS, modified ODI, symptom duration, level of injection, grade of nerve root compression, sacralization	Presence of sacralization may reduce outcomes	≥50% reduction in VAS score	1 hour, 3 weeks, 3 months
23	Kim et al., 2022 [47]	Retrospective	503	L2-3 to L5-S1	Age, sex, pain severity, injection level, lumbar spinal stenosis, HLD	CNN model could help determine outcomes	≥50% reduction in NRS score	2 months
24	Budrovac et al., 2023 [48]	Prospective	59	L5/S1	Age, sex, education, working status, VAS, ODI, disc herniation at one level, nerve contact	Disc herniation without nerve root contact	≥50% reduction in VAS score	1, 3 months
25	Dhandapani et al., 2023 [49]	Prospective	52	L4-5	Age, sex, ODI, pain severity (NRS), single level IVDP, Michigan State University (MSU) grade of IVDP	MSU type 2AB had a lower satisfaction rate	Reduced NRS >50%, and ODI by 40%	24 hours, 1, 3, 6 months
26	Kokar et al, 2023 [50]	Retrospective	260		Age, sex, BMI, NRS, instrumentation, type of ESI, steroid type, medication, complications, minor complications	Short symptom duration and the absence of instrumentation was prognostic factors that positively affect the success of ESI treatment in operated patients	≥50% reduction in pain	1 hours
27	Sariyildiz et al., 2023 [51]	Prospective	286	L4, L5, S1	Age, sex, BMI, disc location, disc morphology, duration of symptoms, injection level, paracentral nerve root compression, Foraminal nerve root compression, preinjection depression	Low-grade nerve root compression, shorter duration of symptoms, extraforaminal/foraminal location predicted better outcomes	≥50% reduction in VAS score	3 months
28	Chalermkitpanit et al., 2025 [52]	Prospective	120	L2-3, to L5-S1	Age, sex, BMI, herniated nucleus pulposus, spinal canal stenosis, level of TFESI, pain severity, symptom duration, classification of disc herniation, location of disc herniation, degree of central canal stenosis, degree of nerve root compression, degree of lumbar foraminal stenosis, spondylolisthesis	Severe central canal stenosis is significantly associated with unfavorable outcomes	≥50% reduction in NRS score	2 weeks
29	Sumen & Kurt Oktay 2025 [53]	Retrospective	21	L4, L5	Age, sex, BMI, level of injection, side of injection, duration of procedure (min), number of shots in fluoroscopy, radiation dose, contrast-spread pattern	Younger age, lower BMI, shorter fluoroscopy duration, and lower dose are significant predictors of success	Successful in 81% patients (pain relief)	Post procedure
30	Jain et al., 2022 [54]	Retrospective	116	L3-4, to L5-S1	Age, sex, BMI, duration of symptoms, neuropathic character of pain, dermatomal distribution of pain, claudication distance, response to anti-neuropathic medication, nerve root compromise	Leg pain which is dermatomal in distribution, responds to anti-neuropathic medications and has Pffirrmann grade 2or 3 in MRI	≥50% pain relief	3 months

**Table 2:** Examined predictive factors of TFESI success TFESI: Transforaminal epidural steroid injection

Category	Factors Studied	Significant Predictive
Patient Characteristics/Clinical Findings	Age, sex, BMI, symptom duration, cause of pain, VAS (visual analog scale), ODI (oswestry disability index), bai (beck anxiety inventory), BDI (beck depression inventory), smokers, worker’s compensation benefits, employment type, preinjection depression, medication used before the treatment, duration of medication use, history of physical therapy, previous spine surgery	Age, sex, BMI, symptom duration, employment type, previous spine surgery, VAS (Pain score), ODI, preinjection depression (worst outcome)
Physical Exam Findings	Sensory deficit, abnormality of reflex, motor deficit, sciatica	Sensory deficit, sciatica
Imaging Findings	Type of disc degeneration (protrusion, extrusion, sequestration), hydration (signal intensity), Spinal stenosis/HIVD (cause), location of herniation (central, left/right central, subarticular, foraminal, or extraforaminal), volume of HIVD, Grade of nerve root compression, compressive lesion, degree of neural compression, high-intensity zone (HIZ), nerve root impingement (NRI), fat infiltration, MSU (Michigan State University) grade of IVDP, Modic changes	HIVD, location of herniation, grade of nerve root compression, Degree of neural compression, HIZ, NRI, MSU grade of IVDP (grade 2AB), Modic changes
Radiologic Characteristics	End plate osteophytes, facet hypertrophy, ligamentum flavum hypertrophy, spondylolisthesis, foraminal narrowing, facet tropism, needle position, direction of contrast flow, level injected, injection type, contrast pattern type, additional extra-epineural injection, instrumentation, duration of procedure, radiation dose	Facet tropism, contrast pattern type, duration of procedure, radiation dose
Anatomical Variances	Sacralization, LS transitional vertebra	Sacralization, LS transitional vertebra
EMG (Electrodiagnostic) Findings	PSW/Fib related to limb muscle LS radiculopathy by EDx, L5 PM score, PM score	PSW/Fib related to limb muscle LS radiculopathy by EDx, L5 PM score
Other	Douleur Neuropathique 4 Questionnaire (DN4) for Neuropathic Pain, TFESI approach (1 level or 2 level)	Douleur Neuropathique 4 Questionnaire (DN4) for Neuropathic Pain, TFESI approach (1 level or 2 level)

Results

**Figure 3:**
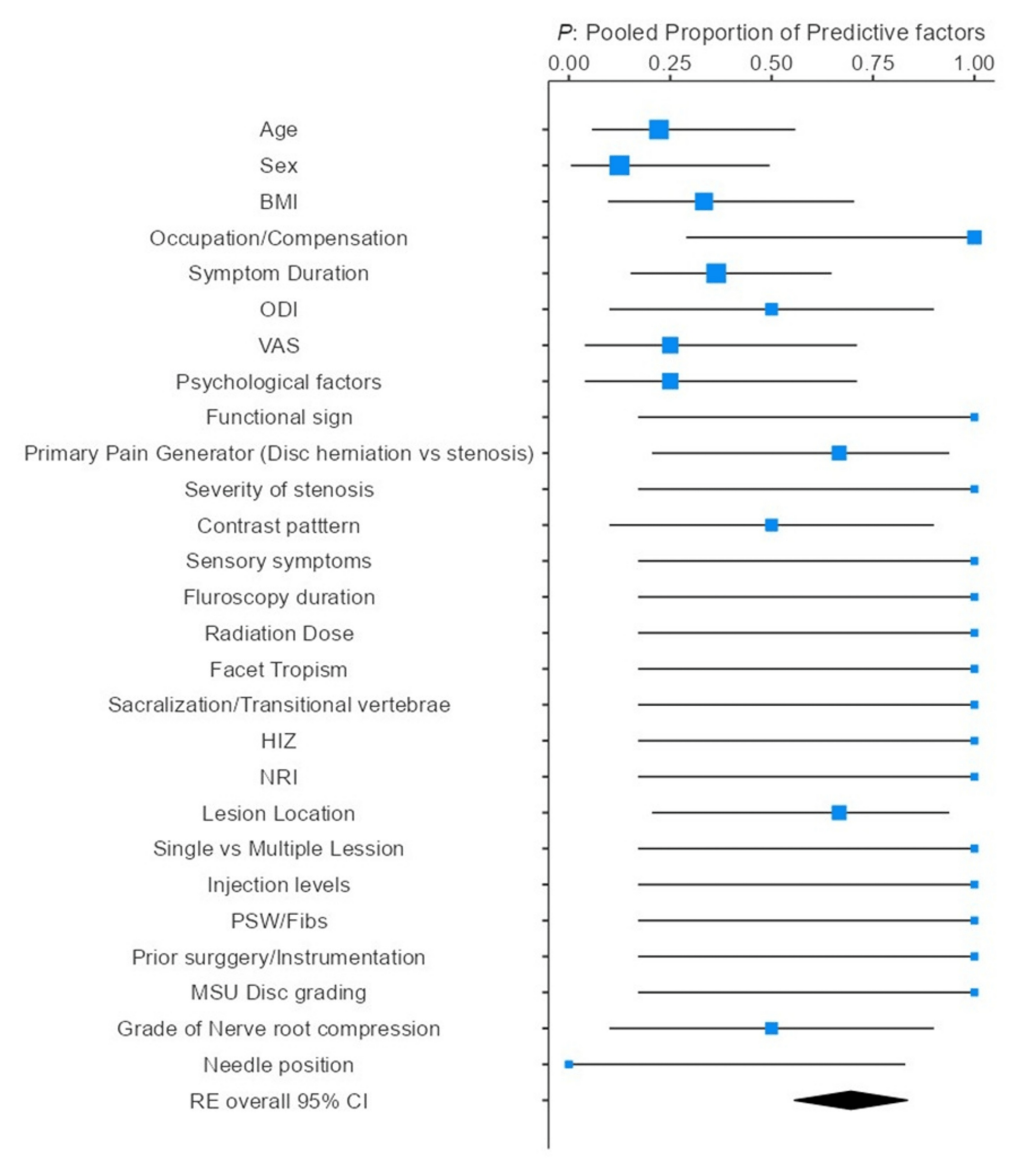
Forest plot showing the proportion of studies identifying each factor as a significant prognostic predictor (k/n), with corresponding 95% confidence intervals

A total of 30 studies were included in this meta-analysis, comprising 14 prospective and 16 retrospective designs, with a combined sample size of 4,346 participants. The studies were published between 2006 and 2025. Across these, 56 prognostic factors were analyzed under seven broad categories, with many studies examining more than one factor. Proportional analysis, pooled effective size, and heterogeneity are presented in Tables 3-5, and forest plots using a random-effects model in Figure 3. The most commonly assessed predictive factor was MRI findings related to radicular pain, particularly disc herniation characteristics and nerve root compression (n=13). Other frequently evaluated factors included age (n=8), symptom duration before injection (n=8), and sex (n=5).

**Table 3:** Summary proportions of studies identifying significant prognostic predictors (k/n) with 95% CI BMI: Body Mass Index, ODI: Oswestry Disability Index, VAS: Visual Analog Score, HIZ: High Intensity Zone, NRI: Nerve Root Impingement, PSW: Positive Sharp Waves, Fibs: Fibrillation Potentials, MSU: Michigan State University

		95% CI	
Study label	P	LL	UL
Age	0.222	0.0568	0.559
Sex	0.125	0.005	0.495
BMI	0.333	0.09636	0.704
Occupation/Compensation	1	0.28947	1
Symptom Duration	0.364	0.15208	0.648
ODI	0.5	0.09992	0.9
VAS	0.25	0.03953	0.71
Psychological factors	0.25	0.03953	0.71
Functional sign	1	0.17059	1
Primary Pain Generator (Disc herniation vs stenosis)	0.667	0.20483	0.938
Severity of stenosis	1	0.17059	1
Contrast pattern	0.5	0.09992	0.9
Sensory symptoms	1	0.17059	1
Fluoroscopy duration	1	0.17059	1
Radiation dose	1	0.17059	1
Facet tropism	1	0.17059	1
Sacralization/Transitional vertebrae	1	0.17059	1
HIZ	1	0.17059	1
NRI	1	0.17059	1
Lesion location	0.667	0.20483	0.938
Single vs multiple lesions	1	0.17059	1
Injection levels	1	0.17059	1
PSW/Fibs	1	0.17059	1
Prior surgery/Instrumentation	1	0.17059	1
MSU disc grading	1	0.17059	1
Grade of nerve root compression	0.5	0.09992	0.9
Needle position	0	0	0.829

**Table 4:** Pooled effect sizes and confidence intervals for significant prognostic factors Note. Estimate is based on a random effects (RE) model.

		95% CI		
Effect	P	LL	UL	k
Pooled proportion of predictive factors	0.696	0.556	0.835	27

**Table 5:** Effect size heterogeneity of significant prognostic factors

			95% CI	
Measure	Level	Estimate	LL	UL
Diamond Ratio	Overall	1.969	1.4972	2.567
H^2^		3.7437	2.1452	6.424
I^2^(%)		73.2886	53.3836	84.433
Τ		0.3122	0.2017	0.439
Τ^2^		0.0975	0.0407	0.193

The follow-up periods ranged from 2 weeks to 2 years, with a median duration of 3 months and a mean of 5 months. All injections were administered under image guidance, most commonly fluoroscopic guidance (n=25), followed by C-arm fluoroscopy and CT guidance in a few studies. The most frequently used corticosteroids were triamcinolone (n=12), methylprednisolone (n=7), betamethasone (n=7), and dexamethasone (n=4), with two studies not specifying the type or multiple steroids. Local anesthetics included bupivacaine (n=16) and lidocaine (n=12), with one study not reporting the anesthetic used. The total injectate volume ranged from 1 to 5 mL.

Patient Characteristics

Across the studies, common demographic factors such as age, sex, and BMI were frequently assessed. Most studies reported no significant associations between these characteristics and outcomes following lumbar transforaminal epidural steroid injection (LTFESI). However, a few studies (22%) identified younger age (95% CI: 0.06-0.56), female sex 12% (95% CI:0.01-0.50), and lower BMI 33% (95% CI:0.10-0.70) as positive predictors of pain relief. Employment status and workers’ compensation were also evaluated; while several studies found no predictive value 100% (95% CI:0.29-1.00), one study suggested that individuals in white-collar jobs experienced better outcomes [36]. Smoking status and previous spine surgery generally showed no consistent association, though the absence of prior spine surgery was noted as a favorable factor in one study [39].

Clinical Findings

The duration of symptoms prior to the procedure was a commonly studied variable. Several studies demonstrated that shorter symptom duration was associated with better treatment outcomes [25,39,50,51], 36% of studies (95% CI:0.15-0.65). However, this was not a consistent finding across all research. When it came to pain severity (measured via VAS or NRS), most studies concluded that baseline pain intensity was not a reliable predictor of post-injection outcomes, though one study reported that higher baseline pain correlated with greater relief (95% CI:0.4-0.71) [33]. Additionally, pre-injection depression was linked to worse outcomes in one study [42], whereas others found no significant impact of psychiatric factors, including depression, anxiety, or somatization on the success of LTFESI (95% CI: CI:0.4-0.71) [34,42,51].

Physical Examination Findings

Findings such as positive straight leg raise (SLR), sensory deficit, motor weakness, and reflex abnormalities were inconsistently reported as predictors. One study found that the presence of subjective paresthesia and objective anesthetic zones significantly predicted better outcomes [36]. Additionally, specific radiculogram patterns, such as the splash type, correlated with favorable results [36].

Imaging Findings

Several radiologic features were identified as prognostic indicators. Low-grade nerve root compression was consistently associated with better clinical outcomes [26,28,51] in 50% of studies (95% CI:0.1-0.90). Disc herniation [34] and herniation without nerve root contact [48] are associated with better outcomes. The location of disc herniation also influenced results, with foraminal, extraforaminal [32,51], and centrally [26] located herniations linked to better pain relief (95% CI:0.20-0.94). Other relevant imaging markers included disc herniation type, volume, and signal intensity, though results varied. Severe central canal stenosis, on the other hand, was associated with poorer outcomes [52], while the RD approach has been significantly associated with better outcomes in patients with severe central and foraminal spinal stenosis [43]. High-intensity zone (HIZ) and nerve root impingement (NRI) demonstrated predictive value at specific time points (95% CI:0.17-1.00) [35]. Modic changes, facet tropism, and fat infiltration were also evaluated, with facet tropism negatively impacting success (95% CI:0.17-1.00) [40]. 

Anatomical Variants

Anatomical differences such as the presence of lumbosacral transitional vertebrae (LSTV) or sacralization were considered in some studies [36,46] (95% CI:0.17-1.00). Both were associated with reduced treatment effectiveness, suggesting that these variants may interfere with proper needle positioning or drug dispersion.

Electrodiagnostic (EMG) Findings

Few studies included EMG-based variables. One study identified that positive PSW/Fib findings in limb muscles were associated with radiculopathy and predicted better short-term pain relief (95% CI:0.17-1.00) [39], highlighting a potential role for electrodiagnostic testing in patient selection.

Radiologic Characteristics of the Injection

Factors like contrast spread pattern, needle position, and direction of contrast flow were examined. While some studies reported no significant predictive value [37] (95% CI:0.17-1.00), specific contrast patterns (Types I and III) were associated with improved pain relief in others (95% CI:0.17-1.00) [29]. In one study, younger patients with shorter fluoroscopy duration and lower radiation exposure had better outcomes, suggesting procedural precision may influence results (95% CI:0.17-1.00) [53].

Discussion

This meta-analysis reviewed 30 studies to evaluate the prognostic factors influencing clinical outcomes following TFNB in patients with lumbosacral radiculopathy. A wide array of variables, spanning demographic, clinical, imaging, anatomical, electrodiagnostic, radiologic, and physical examination domains, were assessed for their predictive value. The findings highlight several consistent trends while also exposing areas of heterogeneity and uncertainty within the current literature.

Across the included studies, patient demographic characteristics such as age, sex, and BMI were among the most frequently examined. Although many studies found no statistically significant relationship between these factors and outcomes, some evidence suggested that younger age [53], female sex [31], and lower BMI may be associated with better post-injection pain relief [31,53]. In particular, lower BMI and younger age were independently correlated with improved results in studies evaluating technical aspects of the injection procedure, such as fluoroscopy time and radiation exposure. Socioeconomic indicators such as employment type also showed relevance [33], with white-collar workers achieving more favorable outcomes compared to manual laborers [36]. While smoking status and workers’ compensation were often examined, their predictive value was generally low or inconsistent [33,36].

Among clinical variables, shorter duration of symptoms emerged as one of the most robust predictors of a positive response to TFNB [25,39,50,51]. This aligns with the general understanding that early intervention during the acute phase of radicular symptoms may be more effective in interrupting pain chronification. Findings regarding baseline pain intensity were mixed; although two studies reported no association between initial pain severity and treatment response [25,36], another study suggested that higher baseline pain scores may predict greater relief [42]. Psychiatric comorbidities, particularly depression, were inconsistently associated with outcomes. One study reported worse outcomes in patients with high pre-injection depression scores [42], whereas others found no significant correlations with anxiety, somatization, or depressive symptoms [29,42,51].

Imaging characteristics provided some of the strongest predictive indicators. Notably, low-grade nerve root compression [26,28,51] and foraminal or extraforaminal disc herniation [32,51] were consistently associated with better outcomes. Conversely, patients with severe central canal stenosis tended to have poorer results [52]. Structural variables such as Modic changes, facet tropism, and disc morphology were inconsistently reported [40] but may also play a role in individual cases. The presence of HIZ and NRI was a time-specific predictor of pain relief [35], suggesting that imaging features may have temporal significance in predicting response.

Anatomical variants such as lumbosacral transitional vertebrae and sacralization were associated with lower success rates [36,46], potentially due to altered biomechanical loading or technical challenges during injection. Similarly, electrodiagnostic features, particularly the presence of positive sharp waves or fibrillation potentials (PSW/Fib) [39], were linked to radiculopathy and may offer additional prognostic value, though further studies are needed in this area.

From a procedural and radiologic standpoint, some evidence suggests that contrast spread patterns and needle positioning influence pain relief, with specific contrast types (Type I and III) associated with better outcomes [29]. However, other studies reported no predictive value of contrast flow direction or needle trajectory [37]. A novel finding from a recent study suggested that shorter fluoroscopy time, lower radiation dose, and fewer shots were predictive of better results [53], underscoring the potential impact of technical precision.

Finally, physical examination findings, including sensory symptoms, motor deficits, and SLR positivity, yielded mixed results. However, certain sensory abnormalities and radiculogram patterns, such as the splash type, showed predictive value in at least one large cohort, suggesting a possible role for detailed neurologic examination and imaging correlation in patient selection [36]. Across studies, a limited number of robust indicators, including "shorter duration of symptoms and low-grade nerve root compression," significantly differentiated individuals who achieved favorable surgical results following TFESI. This encompassed a reduced duration of symptoms and imaging findings indicative of nerve-root compression corresponding to the clinical level, both of which persisted consistently across methodological variations. Conversely, numerous weak or inconsistent predictors, including demographic variables, baseline pain intensity, and psychosocial factors, exhibited highly variable correlations with outcomes and significantly contributed to the observed heterogeneity, indicating that these characteristics alone are insufficient for guiding decision-making.

The clinical decision-making process for lumbar radiculopathy shows different approaches between pain physicians and neurosurgeons because they have different treatment approaches and expectations regarding outcomes. The interventional pain literature guides pain physicians to use imaging results and technical elements, including contrast spread patterns, needle position, and foraminal versus central lesion location, which have been shown to predict the success of transforaminal epidural steroid injections (Cyteval et al., 2006; Paidin et al., 2011; Lee et al., 2013; Maus et al., 2016) [25,29,30,34]. Neurosurgeons generally emphasize patient- and disease-related determinants such as symptom duration, baseline pain intensity, neurological deficits, and anatomical severity of compression (Ghahreman & Bogduk, 2011; McCormick et al., 2014; Kanna et al., 2018) [28,33,36]. The different approaches show that pain physicians use precise procedural optimization to achieve the best nonsurgical results while neurosurgeons concentrate on disc herniation progression, stenosis severity, and surgical intervention possibilities. Notably, the combination of younger age with shorter symptom duration and disc herniation instead of central stenosis has proven to be an effective indicator of positive results in both pain medicine and surgical patient groups (Park et al., 2008; Tecer et al., 2017; Park et al., 2019) [27,35,39]. Consequently, integrating the technical expertise of pain specialists with the disease-severity framework utilized by neurosurgeons might provide a more comprehensive prognostic model to facilitate patient counseling and collaborative decision-making.

The clinical decision-making process for TFESI heavily depends on cost factors, which are frequently overlooked. The total out-of-pocket cash price for a single TFESI procedure costs between $671 in ambulatory surgery centers and $1,174 in outpatient hospital settings [55]. The cost-effectiveness analysis demonstrates that TFESI provides good value through its improvement of quality-adjusted life years (QALYs), especially for patients with radicular pain who do not respond to conservative treatment [56,57]. The unit costs for transforaminal lumbar or cervical injections in the UK range between £700 and £1,100 [58]. While in India, the healthcare infrastructure and service delivery methods lead to substantial differences in treatment costs. The pain clinic Daradia does not display specific prices on its website, but secondary care hospitals in the area estimate TFESI costs between ₹5,000 and ₹20,000 [59]. The procedure at the premier public hospital in India costs between ₹750 and ₹1,500, which makes it an affordable option compared to both private Indian and international prices.

Limitations and Future Directions

Despite the breadth of data, several limitations must be acknowledged. The included studies varied in design, sample size, and follow-up duration, contributing to heterogeneity. Outcome measures were also not uniformly reported, with various thresholds for pain reduction (e.g., ≥50%, ≥60%, ≥75%) and differing use of scales such as VAS, NRS, and ODI. These discrepancies limit the ability to perform pooled quantitative analysis and necessitate cautious interpretation of results. Moreover, many predictors were only evaluated in isolated studies, making it difficult to draw definitive conclusions regarding their clinical utility.

Future research should aim for standardization of outcome reporting, longer-term follow-up, and inclusion of multi-modal predictors, including clinical, imaging, and psychosocial variables. There is also a growing opportunity to explore machine learning-based predictive models, as demonstrated in one of the included studies, which may enhance individualized treatment planning.

## Conclusions

This meta-analysis synthesizes findings from 30 studies examining 56 prognostic factors in seven broad categories influencing outcomes following LTFESIs. While demographic variables such as age, sex, and BMI were frequently analyzed, their predictive value for treatment success remained inconsistent across studies. Certain clinical factors, including shorter symptom duration and absence of prior spine surgery, showed a more consistent association with favorable outcomes. MRI findings emerged as the most commonly investigated and reliable predictors, particularly characteristics like low-grade nerve root compression and specific disc herniation features (disc herniation, herniation without nerve root contact, and the location of disc herniation (foraminal or extraforaminal, or centrally located)). Anatomical variations, such as lumbosacral transitional vertebrae, and procedural variables like contrast spread patterns and fluoroscopy precision, also influenced treatment efficacy. Overall, this review highlights the multifactorial nature of outcomes following LTFESI, emphasizing the need for a comprehensive patient evaluation incorporating clinical, radiological, and procedural parameters to better guide treatment decisions and improve patient selection.
